# Technological advances enabling an enhanced understanding of early Alzheimer's disease

**DOI:** 10.1002/alz.70883

**Published:** 2025-11-02

**Authors:** Rhoda Au, Niranjan Bose, Farhad Imam, Vijaya B. Kolachalama

**Affiliations:** ^1^ Department of Anatomy & Neurobiology, Neurology and Epidemiology Boston University Chobanian & Avedisian School of Medicine Boston Massachusetts USA; ^2^ School of Public Health Boston Massachusetts USA; ^3^ Department of Medicine Boston University Chobanian & Avedisian School of Medicine Boston Massachusetts USA; ^4^ Gates Ventures Kirkland Washington USA; ^5^ Global Research Platforms LLC Kirkland Washington USA; ^6^ Department of Computer Science and Faculty of Computing & Data Sciences Boston University Boston Massachusetts USA

**Keywords:** Alzheimer's disease, artificial intelligence, data science, dementia, digital health technologies, early detection, machine learning, technologically‐enabled scientific innovation

## Abstract

The aspirations of Alzheimer's disease (AD) precision medicine and precision brain health are not fully realized despite significant advances in early pathological detection. Hampering progress is the difficult challenge of the accurate detection of behavioral symptoms that can help differentiate those at high risk specific to AD versus those with more AD and related dementias (ADRD), who may have AD along with other types of dementia, as well as those who will and will not progress to clinically expressed disease. Technological advances in hardware, software, and analytics are paving the way to opportunities to overcome these long‐standing barriers to early clinical symptom detection, when recent and emerging interventions might be most effective. It is important to note that these innovations must not exist in isolation. Integration of data from both AD/ADRD related studies and non‐AD related studies is needed whenever brain‐related measures are collected to fully realize the scientific opportunities. We envision a near future of harmonized participant data sharing, encompassing standardized multimodal data alongside emerging digital streams, to deliver an invaluable resource for the global research community.

**Highlights:**

- Technology is overcoming barriers to accurate early detection of behavioral symptoms of AD/ADRD.
- The National Alzheimer's Coordinating Center is paving the way for the integration of technological advances into AD/ADRD research.
- A technologically‐enabled framework provides a solution for open science on a global scale.

According to the World Health Organization, 2020 marked a significant shift in world age demographics, in which people 60 years of age or older outnumbered individuals younger than age 5.^1^ These aging trends have highlighted Alzheimer's disease (AD) and related dementias (ADRD) as a global epidemic. It is worth noting that AD refers to those with pathological findings specific to AD (e.g., beta amyloid plaques, tau neurofibrillary tangles), while ADRD is representative of a broader range of dementias that include AD but also other pathologies that also contribute to risk of AD but are not specific to AD (e.g., vascular dementia, lewy body dementia, frontotemporal dementia). Going forward ADRD will be used to represent both AD specifically and the broader category of ADRD. Within the United States, a rapidly growing aging population has led to a steady rise in the number of those diagnosed with ADRD,^2^ despite evidence that incidence rates of clinically diagnosed disease are declining because of better management of ADRD risk factors, such as those also associated with cardiovascular disease and higher levels of educational attainment.^3^, ^4^

In recognition of the fact that ADRD remains an urgent health care priority, in October 2024, the United States Congress renewed the National Alzheimer's Project Act (NAPA), which was initially signed into law in 2011. The context has changed dramatically since the NAPA was signed, with over two decades of research progress that has been led by many of the National Institute on Aging (NIA)–funded Alzheimer's Disease Research Centers (ADRCs) across the United States, including several U.S. Food and Drug Administration (FDA)–approved anti‐amyloid drug treatments and a promising pipeline of Phase 1–3 clinical trials.^5^ Furthermore, increasing awareness that ADRD is a life‐course disease with many etiological pathways has led to greater emphasis on brain health as a prevention strategy, which will further reduce AD/ADRD incidence. Despite these positive directions for treatment and prevention, other studies have revealed that AD is often comorbid with other neurodegenerative pathologies, and clinical expression is highly variable across an insidious onset timeline that can take decades to reach clinical diagnosis thresholds. Thus, the concept of precision medicine, which aspires to determine the right treatment for the right person at the right time, has become the key to defining NAPA's future success in achieving effective treatment. We contend that technological advances can bring forward the opportunity to change the trajectory of symptoms through even earlier invention, resulting in a precision brain health vision for enabling disease prevention.

Neuropsychological tests have long been the standard for measuring cognition, the hallmark behavioral symptom of ADRD. But many neuropsychological tests are highly biased, with performance mediated by education, language, culture, and sex/gender. Other factors also impact neuropsychological test performance at the time of testing, such as sleep, mood, and environmental interruptions. The resulting fluctuations in performance make it difficult to distinguish a person's normal range of daily fluctuations from changes reflective of neuropathological onset and progression. Furthermore, other ADRD behavioral symptoms that also impact cognition (e.g., mood, orientation, and sleep) have similar frequent undulating patterns that make discernment of an emerging underlying pathological cause a complex problem. Because research has long demonstrated that ADRD pathology can be evident in the absence of clinical symptoms, current drug treatment decisions are now highly dependent on the earliest detection of cognitive or other clinical indicators of disease. Preventive interventions will also depend on precision monitoring to demonstrate effectiveness. The ability to detect subtle signals of cognitive change must also be tempered with caution to avoid overdiagnosis. Not every deviation from a normative curve implies pathology, and not every biomarker‐positive individual should be treated. A future of precision medicine and precision brain health must differentiate between disease risk and disease presence. The present quandary is how to do so with the level of precision that is necessary.

Technological advances that have been moving in parallel with scientific ones provide solutions for the next stage of ADRD research. Although the use of computerized testing of behavioral symptoms, particularly that of cognition, have long been in existence,^6^, ^7^ these assessments are no more accurate than paper‐and‐pencil tests. In his prescient 2008 paper, Dr. Jeffrey Kaye laid the foundation for applying technology to monitor highly variable AD/ADRD‐related behaviors,^8^ where he envisioned a sensor‐based home environment to monitor longitudinal fluctuations in the home over many years. He followed that with an initial study to test the efficacy of home‐based cognitive assessments, which identified both barriers for technology use by older participants and the potential promise of these tools if familiarity were established.^9^ In collaboration with other colleagues, Dr. Kaye led the first study to demonstrate how home‐based assessment were feasible for assessing AD/ADRD‐related symptoms.^9^ For example, embedded sensors in the home could monitor for changes in gait^10^ and walking speed^11^ and sleep patterns,^12^ and these observations could be done longitudinally and unobtrusively.^13^, ^14^ As the hardware continued to evolve, Kaye and colleagues continued to incorporate these emerging technologies to addressing problems such as interactive robotics,^15^ monitoring of computer use, and responses to online questionnaires to better track much more subtle changes in function^16^, ^17^, ^18^, ^19^ and driving behavior^20^ as an alternative to overt assessment methods.^13^, ^21^, ^22^, ^23^, ^24^ Others soon followed suit, applying the home sensor‐based system, Collaborative Aging Research Using Technology (CART),^25^ to more diverse home environments^26^, ^27^ and leveraging existing home‐based^28^ and mobile technologies.^29^

The penetration of internet‐of‐things (IoT) consumer grade mobile devices, including tablets, smartphones, and wearables, has fueled the sea change in the application of technologies to accurately detect the earliest changes in highly heterogeneous cognitive and other behavioral symptoms, and to monitor them more frequently. Globally, there are ≈7.2 billion (B) smartphones and an estimated 41.6B digitally connected devices worldwide.^30^ Given the rise of digital data collection tools, it is now possible to achieve population‐level breadth alongside deep clinical characterization, translating into meaningful advances in both research and clinical utility.

Although much of the focus of precision AD medicine centers around molecular or ‘omics profiling^28^, ^29^ to identify biological pathways and mechanisms of AD and other often comorbid neurodegenerative pathologies, equally important is the need to track biological disease indicators with clinical expression. This reinforces the earlier point that evidence of AD biomarkers does not always lead to clinical expression of disease and raises two practical considerations that are laid out below.

First, there is abundant evidence that current AD research data is fraught with representative gaps, both within the United States and globally. These gaps mean that the extent to which AD biomarkers manifest themselves clinically is still largely unknown. For example, since the 1990s, validation of in vivo AD pathology has relied on imaging biomarkers, initially with non–AD‐specific magnetic resonance imaging (MRI) scans^31^, ^32^, ^33^ that later gave way to AD‐specific positron emission tomography (PET) scans^34^ that used tracers of amyloid and tau, both of which have recently received FDA approval because of their high levels of biomarker specificity.^35^ Clinical studies and trials have adopted PET scans as in vivo evidence of AD pathology, which greatly limits population reach not only due to the high costs of imaging, but also because PET facilities must be located near a cyclotron, as the half‐lives of amyloid and tau tracers are ≈110 min.^34^, ^36^ This logistical constraint severely restricts accessibility in rural or underserved areas, thereby reinforcing geographic and socioeconomic disparities in biomarker‐driven AD research and care. Fluid AD biomarkers are solving the limitations of PET scans. Cerebrospinal fluid (CSF) is recognized as the gold standard method for identifying in vivo AD pathology,^37^ but lumbar punctures from which to draw CSF are considered too invasive for many participants recruited into AD clinical studies.^38^ Thus, until recently, reliance on these methods for AD confirmation has resulted in studies that are heavily biased by overrepresentation from large, highly resourced, academic‐affiliated hospitals located in densely populated urban cities, where study samples are disproportionately from highly educated people of white European descent. As a result, it remains largely unknown to what extent in vivo evidence of AD biomarker pathology remains clinically silent in people who have different demographic or racial‐ethnic profiles.

Another significant representative gap is tied to the increasing recognition that ADRD are life course diseases, which has gained increasing traction over the last 20 years^39^, ^40^, ^41^, ^42^; the seeds of risk can be sowed beginning in the earliest years of life, with additional risk factors cumulating across the lifespan. Many studies of ADRD, however, oversample for people in the older age ranges at study onset, with ages of 60–65 being the most common. Moreover, blood‐based biomarkers are the much‐heralded solution to overcoming the persistent reliance on methods that are not feasible at large scale. The promise of blood‐based biomarkers is highlighted by Zetterberg and colleagues.^43^ Yet, despite their scalability, blood‐based biomarkers are not immune to limitations including variability across assay platforms, sensitivity to comorbidities, lack of spatial characterization of disease, and the need for contextual interpretation based on disease stage and pretest probability, which must be addressed before widespread clinical adoption.

The second practical concern is the long‐standing challenge of detecting highly heterogeneous clinical symptoms with both high sensitivity and specificity. As noted previously, in vivo evidence of AD pathology does not mean that clinically expressed disease will follow. Studies of cognitive resilience have identified factors that delay or prevent people from reaching clinical thresholds for diagnosis.^44^, ^45^, ^46^ Ethical concerns about treating people asymptomatic for disease with newly approved disease‐modifying therapies, particularly those associated with serious side effects like amyloid‐related imaging abnormalities (ARIA) and high costs, have contributed to the limited uptake of the first FDA‐approved drugs for AD since donepezil (Aricept) and memantine (Namenda). Inexplicably, although much investment has been made in imaging, blood, and genetic/genomic biomarkers of AD, clinical trials still rely largely on clinical assessment tools such as the Clinical Dementia Rating Sum of Boxes (CDR‐SB)or the Alzheimer's Disease Assessment Scale‐Cognitive (ADAS‐Cog), developed in 1982^47^ and 1997,^48^ respectively. The conundrum that has been perpetuated even amid significant scientific advances is the continued use of these blunt instruments, administered sporadically, to measure outcomes of much earlier biological markers of the disease. To resolve these challenges, the network of ADRCs within the United States has been expanding, with the goal of serving as a central resource across the country for promoting and conducting clinical studies and trials, as well as facilitating implementation in clinical care settings. Each ADRC provides an infrastructure for integrating technological solutions to address many of the gaps identified above.

## 1 DIGITAL VOICE AS A NEW FRONTIER

Through the recently launched Uniform Dataset (UDS) protocol, version 4 (UDS 4.0), a new optional digital component has been added to the neuropsychological test protocol. Digital voice recording of spoken responses to the tests is now recommended as a way to increase the potential sensitivity to detecting cognitive changes, without increasing the test burden on participants. These recordings can be accomplished easily through multiple recording options, such as a hand‐held recorder, a tablet, or video‐conference tools. Studies have shown that digital voice recordings can be analyzed to detect cognitively related features, both comparable to neuropsychological tests and as an alternative approach to detecting cognitive impairment.^49^, ^50^, ^51^, ^52^ In addition, analysis of digital recordings has been shown to be predictive of dementia diagnosis up to 6 years earlier.^53^

Of note, since most people speak, biases related to education, language, and culture can be eliminated. Initial studies have also already demonstrated the feasibility of voice as a marker across multiple cohorts that come from the Unites States and other countries with speakers of various languages.^54^, ^55^ Additional preliminary analyses have suggested that acoustic features can be identified that are common across languages, laying the foundation for future work in which cognitively related voice features may lead to assessments that may be more universal than current neuropsychological tests that are being translated into multiple languages, but still carry inherent education, language, or cultural biases.^56^, ^57^, ^58^, ^59^

## 2 BEYOND DIGITAL VOICE

Digital voice is just the beginning. Harnessing the smartphone's multi‐sensor capabilities has been widely touted^60^, ^61^, ^62^ but not yet realized. Most research using smartphones remains tethered to legacy methods of cognitive assessments, such as converting paper‐and‐pencil–like neuropsychological tests into smartphone applications. Another common practice is converting validated questionnaires that solicit self‐reported ADRD‐related clinical information into smartphone‐ or web‐based applications. Like voice responses, these test‐like applications and questionnaires also capture time‐stamped features that are then converted into derived measures such as item response speed, correctness, and missingness. These data bring a level of granularity that cannot be captured by long‐used assessment instruments.

Today marks an inflection point. The 41.6B IoT options provide the technological infrastructure from which to build a large‐scale digital data collection strategic plan that will fill the representative gaps, including across the lifespan. The ADRCs are poised to be part of this grand vision, with many ADRCs already collecting digital data. The National Alzheimer's Coordinating Center (NACC) has initiated several pilot projects that are testing the infrastructure for them to ingest digital data and integrate with all the other existing clinical and molecular data that they have aggregated across the ADRC network.^63^ The question remains is how to not only use the multi‐sensor digital data collected through the hardware that is already out in the field but also build the data management backend that will be required to translate these advances from the academic arena into clinical trials and patient care.

## 3 RAPID TECHNOLOGICAL ADVANCES IN A NEW ERA OF OPEN SCIENCE

The narrative here shifts from what is known and being done today to what needs to be done to fully realize what technology is meant to do. What follows is a glimpse into a possible framework for the future, one that balances pragmatic goals achievable within the next 3–5 years while remaining sufficiently flexible to adapt or respond to unforeseen developments. Technology lifecycles are notoriously short. A case in point is the frenetic pace by which new artificial intelligent (AI)–driven solutions developed by OpenAI or DeepSeek are upending business as usual and doing so by building tools that can be used by those with limited technical acumen or skills. Research and health care settings are notoriously resistant to swiftly changing course, and for good reason. Implementing ill‐considered practices can have serious ethical consequences and a significant impact on people's health and well‐being. As a result, however, there is currently a significant disconnect between how quickly technologies advance and how slowly research and clinical settings react that is slowing progress on scientific discoveries and advancing better clinical care.

The ADRCs through NACC are at the forefront of open science, which is being promulgated by the National Institutes of Health (NIH). The NACC has initiated several pilot projects that are testing the infrastructure for them to ingest digital data and integrate with all the other existing clinical and molecular data they have aggregated across the ADRC network.^63^ Real‐time data sharing is a key strategy that can break the impasse between out‐of‐sync timelines that are slowing progress.

Open science will be difficult to realize if there is too much reliance on proprietary digital tools and commercial biomarker platforms. Without open validation, reproducibility is difficult to demonstrate. Furthermore, if commercial products are developed focused on lucrative markets, there is a risk of exclusion of under‐resourced communities, further perpetuating already known inequities. The future of ADRD care must be grounded in science that is transparent, accessible, and publicly accountable, and mindful of privacy concerns. Open science needs protocols and the data collected using these protocols to be widely shared. It means building data management infrastructure that meets FAIR standards (Findable, Accessible, Interoperable, Reusable) using open‐source tools so that others may readily build their own and connect them through application programming interfaces (APIs). Open science in the context of technology, means developing the complete front‐to‐backend pipeline that will prevent a digital divide and instead promote equal opportunity science.

**FIGURE 1:**
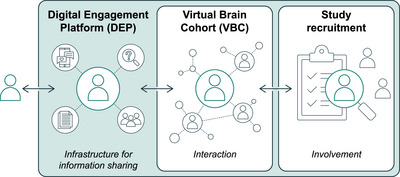
Digital engagement platform. A conceptual framework depicting the progression of user engagement in brain health initiatives, from initial information exchange via knowledge sharing to interactive participation in a virtual brain community, and culminating in active involvement through study recruitment. The underlying digital data stream represents the foundational infrastructure, enabling seamless connectivity and data flow across these stages.

The figures below represent a conceptual framework for a technologically enabled open science ecosystem. It begins with applying well‐developed social engagement platform methods to build a platform centered around brain health (Figure 1). Research studies often include exclusion criteria, which necessarily means turning away those who are interested but for various reasons cannot be included. Within the ADRCs, each center has a specific AD/ADRD theme. For example, the Boston University ADRC focuses on exposure to traumatic brain injury (TBI) and repetitive head impacts (RHIs) as an increased risk for not only AD, but also chronic traumatic encephalopathy (CTE), whereas the University of California San Francisco ADRC specializes in frontotemporal dementia. Collectively across the 35 ADRCs, the multi‐etiological spectrum of AD/ADRD is represented, but at each site, there is limited capacity to enroll everyone interested into their longitudinal cohort. Subject burden and insufficient resources restrict how many participantany single ADRC can include in their study that adheres to the UDS protocol or for any affiliated studies. Recruitment results in excluding people who would like to be in an ADRC study. AD clinical trials have high screen‐fail rates, with upward of 90+% of people who raise their hand not being enrolled. The concept of a brain health–focused digital engagement platform (DEP) creates the opportunity to re‐define “screen‐fail” for a specific study as “screen‐enroll” into either the specific study or into a virtual brain community that is hosted on the DEP. Such an approach could increase engagement with potential study participants as well as overall rates of AD/ADRD research enrollment.

This is a shift from the current practice of study registries, where people screened out for a study reside in relatively scientifically dormant portals. The DEP will act like any social engagement platform, where brain health–related information that is responsive to the participant's specific health‐related interests is provided continuously through precision communication tools. Furthermore, interactive communities are developed through the inclusion of families and friends, centered around the theme of brain health. For those interested in participating in research, the participant along with their family and friends can enroll into a virtual brain cohort study, where data could be collected through a license‐free smartphone application and a bring‐your‐own‐device (BYOD) strategy that capitalizes on the 41.6B IoT devices that are already in use. Over time, as the virtual brain community (VBC) grows, it is anticipated that ancillary projects will emerge that will extend the initial scope of the VBC.

**FIGURE 2:**
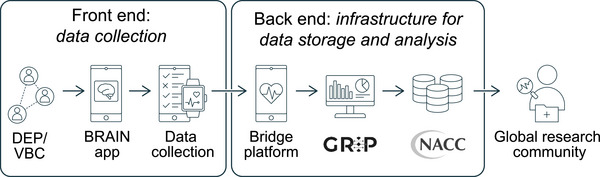
Front‐to‐backend solution. An illustration of the data flow in a digital ecosystem for brain health research, starting from user engagement through a digital engagement platform (DEP) integrating social media tools, progressing to a virtual brain community (VBC) for interactive participation, and extending to data collection via customizable smartphone applications (e.g., “Brain App”) and wearables. This front end is bridged to the global research and imaging platform (GRIP) for secure backend processing, management, and integration of multimodal data streams.

Figure 2 illustrates an envisioned front‐to‐backend solution that includes not only the DEP to build the VBC, but also provides the infrastructure needed to support data collection through a smartphone application. Currently a collaboration between researchers at Boston University and Global Research Platforms (a subsidiary of the Alzheimer's Disease Data Initiative) on the Global Research and Imaging Platform (GRIP)^64^ is developing a smartphone application named Chameleon that collects digital data streams from active engagement tasks and questionnaires and through passive engagement via sensors. Of note, this application is both iOS and Android compatible, customizable both to the native language and local needs of the participant, and will be white‐labeled so that it can be incorporated into and branded to any study. The team is also integrating components of the Bridge platform developed by Sage Bionetworks to provide a digital data management system^65^, ^66^ that includes a dashboard and secure workflow environment. To assist the next step after data acquisition, GRIP, in collaboration with multiple ADRCs, is building open‐source digital voice, imaging, and other data‐processing tools in its modular platform that will allow scientists to pick and choose the solutions that fit their specific needs. Digital data in its raw native format or processed using tools that are available through GRIP can then be integrated into the NACC along with all other ADRC participant data and shared via the NACC Data Front Door, which then makes these data available to the global research community.[Fig alz70883-fig-0003]

**FIGURE 3 alz70883-fig-0003:**
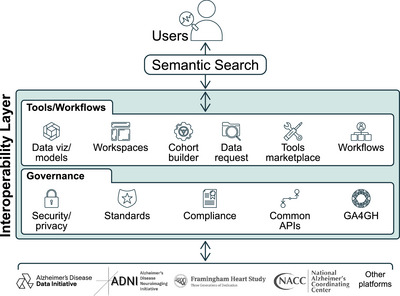
Interoperability across interoperability platforms. An integrated data ecosystem for biomedical research, highlighting the semantic layer enabling advanced search and access with interconnected tools and workflows, ensuring compliance, reproducibility, and extensibility.

In addition to NACC, there are many other existing data repositories that have collected AD/ADRD‐related genetic, multi‐omics, clinical, and in some cases, digital data, and many more being expanded or initiated. Digital platforms such as DEP and smartphone‐based data collection applications will break down costly infrastructure[Fig alz70883-fig-0004] barriers that have limited research in low‐ to middle‐income resourced environments within the United States and internationally. These new digitally enabled repositories can fill representative data gaps (racial/ethnic, income, geographic). However, as more of these data repositories are generated, interoperability between the NACC and these other interoperability platforms (see Figure 3) will be necessary to build a globally representative data resource to shift the aspirations of precision AD medicine and precision brain health to one that can now be realized.

**FIGURE 4 alz70883-fig-0004:**
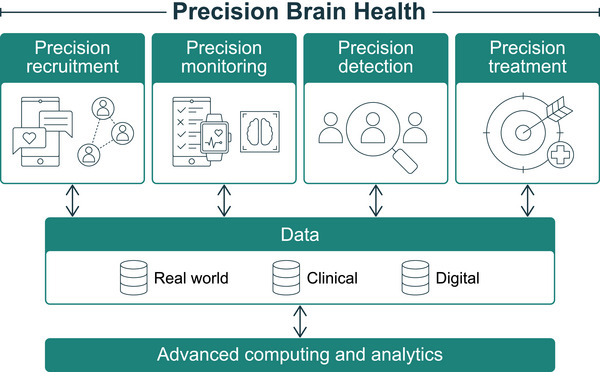
Digital is the new blood. A conceptual framework for precision brain health, illustrating the progression from precision recruitment (leveraging digital networks and social platforms) through precision monitoring and detection (using multimodal sensors for body and brain metrics) to precision treatment (targeted interventions). Supported by diverse data sources, this model underscores how digital data serve as a non‐depleting, reusable resource for ongoing analysis, akin to blood samples, empowered by advanced computing and analytic tools at its foundation.

Since its establishment in 1999, the NACC has become an invaluable ADRD data resource. It currently houses over 200,000 clinical assessments that include the shared UDS data collection protocol common to all ADRCs and additional cognitive, genetics, imaging, fluid biomarker, and neuropathological data that NACC is harmonizing across ADRCs. With the digital voice and other digital modalities now being widely adopted by ADRCs, coupled with rapidly developing data science and AI approaches, NACC is poised to help usher in new era of science. The digital revolution is not limited to digital capture of traditional neuropsychological paper‐and‐pencil tests that were the mainstay of UDS 1.0–3.0. Nor is it limited to cognition. Digital data in its native format are not dissimilar to blood collected in a test tube, waiting to be reanalyzed for multiple clinical, genetic, and multi‐omics markers that are relevant across multiple diseases. Although each sensor stream offers unique insights, the true power of a future vision of digitally enabled precision medicine and precision brain health lies in multimodal integration, a challenge that remains largely unfulfilled. Harmonizing data across voice, mobility, sleep, and device interactions requires rigorous computational modeling, interpretability, and clinical relevance. The Framingham Heart Study, the UK biobank, and the Health and Retirement Study are examples of data repositories that are being used by researchers worldwide to study many diseases of interest. Digital data, unlike blood data, are a non‐depleting resource that can be reused and reanalyzed an infinite number of times. As illustrated in Figure 4, digital data can lead to a new frontier. Technology integration into the scientific enterprise should not be done simply to do a better version of what is currently being done. To fully leverage technologies available today and continue to do so through the technologies that are continuously emerging requires embracing a shift in mindset from the more static methods of the past to a much more fluid and dynamically changing research infrastructure that cuts across the entire research enterprise. Capturing the data generated from billions of IoT devices will allow reach to many who have not been represented previously. The sensors embedded in these devices will collect longitudinal data at a much higher rate of frequency leading to much more accurate monitoring of symptoms that are highly heterogeneous and the detection of subtle changes that are nonetheless clinically meaningful. Accurate early detection sets the stage for treatment much earlier in the course of an insidious onset process that is not just characteristic of ADRD but of virtually all major chronic diseases. Furthermore, the etiological pathways for ADRD share those with other chronic diseases. For example, detection of a persistent increase in systolic blood pressure (SBP) that can be done through smartphone applications and wrist worn wearables, can lead to lifestyle interventions to attenuate SBP increases and even reverse SBP levels back to earlier baseline levels, thereby decreasing not only the future risk of ADRD but also heart disease and stroke. Multi‐sensor monitoring that is continuously detecting changes across multiple ADRD risk factors, resulting in combination therapies whose net results leads to fewer risk factors reaching disease thresholds, is the precision brain health vision that a newly envisioned technologically‐driven research enterprise could enable. Underlying this bold vision will be the need for developing better, more automated, and widely available digital data processing tools.

Advanced data science and AI analytics play an important role here, enabling the extraction of actionable insights from vast, heterogeneous datasets through techniques like machine learning for predictive modeling of disease progression, deep learning for pattern recognition in multimodal data, and AI‐driven multi‐omics integration for early diagnosis and personalized interventions. Looking further ahead, quantum computing emerges as a speculative yet intriguing frontier, with potential applications in simulating complex molecular interactions for drug discovery, optimizing quantum‐enhanced AI models for neurodegeneration pattern analysis, or accelerating quantum machine learning for early AD screening through hybrid classical‐quantum frameworks. However, its practical promise remains undetermined, constrained by current hardware limitations, scalability challenges, and the nascent stage of real‐world implementations in biomedical research.

## 4 CONCLUSION

The convergence of an aging global population, scientific progress in ADRD plasma biomarkers, and the ubiquity of digital technologies marks a defining moment for precision medicine and precision brain health. Yet realizing this potential demands more than just scientific or technological innovation. It requires a fundamental shift in how we design, manage, scale, and democratize access to assessment tools, data infrastructure, and clinical pathways. We must move beyond legacy instruments and build a future‐ready ecosystem grounded in open science, ethical transparency, and inclusive design. Through the NACC, the ADRCs and the broader research community are uniquely positioned to lead this transformation. By leveraging smartphone‐based sensing, interoperable FAIR‐compliant platforms, and multimodal analytic approaches, we can expand reach, reduce bias, and bring the promise of precision AD/ADRD prevention and care to all. The time to act is now, precisely before opportunity lags the need, and progress is constrained by inertia.

## CONFLICT OF INTEREST STATEMENT

R. A. is a scientific advisor to Signant Health and NovoNordisk. V. B. K. is on the scientific advisory board for Altoida, Inc., and is a co‐founder of deepPath, Inc., and Cognimark Inc. N. B. and F. I. have nothing to disclose. Author disclosures are available in the supporting information.

## Supporting information



Supporting information
